# Risk Categorization in On-Farm Welfare in Different-Sized Dairy Sheep Flocks

**DOI:** 10.3390/ani14101401

**Published:** 2024-05-07

**Authors:** Federica Salari, Cristina Roncoroni, Francesco Mariottini, Alessandra Muzic, Iolanda Altomonte, Irene Sodi, Susy Creatini, Lorella Giuliotti, Giovanni Brajon, Mina Martini

**Affiliations:** 1 Department of Veterinary Science, Università di Pisa, Viale delle Piagge 2, 56124 Pisa, Italy; federica.salari@unipi.it (F.S.); alessandra_muzic@yahoo.it (A.M.); irene.sodi@phd.unipi.it (I.S.); susy.creatini@phd.unipi.it (S.C.); lorella.giuliotti@unipi.it (L.G.); mina.martini@unipi.it (M.M.); 2 Istituto Zooprofilattico Sperimentale del Lazio e della Toscana “M. Aleandri”, Via Appia Nuova 1411, 00178 Rome, Italy; cristina.roncoroni@izslt.it; 3 Istituto Zooprofilattico Sperimentale del Lazio e della Toscana “M. Aleandri”, Via Castelpulci 43, 50018 Florence, Italy; francescomariottini93@gmail.com (F.M.); giovanni.brajon@izslt.it (G.B.)

**Keywords:** dairy sheep, animal welfare, ClassyFarm, farm management, animal-based measures

## Abstract

**Simple Summary:**

Sheep farmers face increasing pressure to produce more in order to meet market demands, while consumers have more interest in animal welfare status and the environment. The aim of this paper was to categorize the on-farm welfare risks using the ClassyFarm protocol and to identify if welfare risk changed according to farm size. The critical issues we found were the low number of stockpersons, the small areas available for the animals, the lack of udder cleaning procedures during milking, and the lack of prevention plans for the most important infectious ovine diseases. As the number of animals within the farm increased, the management of the flock improved; on the other hand, the number of animal inspections decreased and the hygiene of the water worsened.

**Abstract:**

The objective of the present work was to categorize the risks in the on-farm welfare of dairy sheep in semi-extensive systems in order to highlight if risks in welfare vary according to the farm size. To the best of our knowledge, this study constitutes one of the few categorizations of the risks in the welfare of dairy sheep reared semi-extensively. The survey was conducted on 12 semi-extensive dairy sheep farms in Tuscany (Central Italy), which were classified according to flock size: <500, from 500 to 1000, and >1000. The results showed an adequate rating for all the farms included in the study. The most critical issues concerned the ratio between the number of stockpersons and the number of animals within the farms, the small decubitus areas, the lack of udder cleaning procedures during milking operations, and, in terms of biosecurity, the lack of prevention, control, and eradication plans for the most important infectious ovine diseases. The results show that different items in the five areas evaluated, including the assessments of management of the flock, the number of daily inspections, and the hygiene of the water supplies are associated with the farm size. In large farms, the assessment of management of the flock was better, but the number of daily inspections and the hygiene of the water supplies were worse.

## 1. Introduction

The dairy sheep sector in Europe contributes to the production of many typical dairy products of interest for the international market [1]. Currently, farmers and processors face increasing pressure for production intensification in order to meet market demands, while, on the other hand, they need to address consumer expectations for high animal welfare status and environmentally friendly management practices. The European Union requires national authorities to provide control plans based on specific indicators for each species and type of farming, aimed at ensuring a high level of animal welfare, as well as food labeling to transmit value throughout the food chain [2]. The use of a tool to assess animal welfare is advantageous for farmers, as it can provide them with useful information on structural and managerial improvements. Nevertheless, there are few data on dairy sheep welfare status, especially compared to pigs, poultry, and dairy and beef cattle mostly in intensive farming systems [3].

In recent years, different systems for evaluating animal welfare on farm have been developed [4] founded on animal-based and/or management- and resource-based indicators. Animal-based indicators are direct indicators of the welfare of the animal, while management- and resource-based indicators (such as nutrition management or provision of shelter) are also relevant for more comprehensively assessing the welfare issue [5]. ClassyFarm [6] is an officially recognized system using animal-, management-, and resource-based indicators in order to categorize risks on livestock farms, and was developed by the National Reference Centre of animal welfare, funded by the Italian Ministry of Health. Using a specific checklist, the ClassyFarm protocol is aimed at supporting official controls, collecting data, promoting the implementation of welfare levels, and providing consumers with information.

In the Mediterranean area, the dairy sheep sector is characterized by semi-extensive farms using natural pastures as the main feeding source. This feature, as well as the low entrepreneurial approach and the large variety of farming types, make the welfare assessments of sheep challenging [7].

Semi-extensive systems are often considered more natural and closer to the ethological needs of the animals. In fact, grazing is a natural behavior of ruminants that ensures the possibility of movement and interactions with other conspecifics [8]. This is in line with public opinion, which associates the image of the grazing animal with ideal conditions. In addition, grazing positively influences the animal’s metabolism, as well as activating a greater elimination of plasma triglycerides and promoting animal health and longevity [9]. However, grazing sheep are also less controlled, as they spend part of the day outdoors and return to the fold only at night, and the natural environment can have a negative impact on animal welfare by hindering the animal’s ability to cope with challenges [10]. Dairy sheep living in extensive or semi-extensive farming conditions can have problems with maintaining an appropriate level of welfare, mostly concerning climatic variations, the availability of adequate food and water, enteroparasites, and predation [7].

In addition, the average size of dairy herds has continuously increased over recent decades in all developed countries [11]. In particular, in the sheep sector there has been a reduction in the number of farms and a marked increase in the number of animals per flock [12]. The herd size has been associated with increases in stocking density and stock per labor unit, and this could be hypothesized to increase the risk of adverse animal welfare outcomes [13]. To the best of our knowledge, there are few studies on dairy sheep examining risks in welfare in relation to farm size. This study constitutes one of the few categorizations of the risks in the welfare of dairy sheep reared semi-extensively. The aim of this paper was to categorize the on-farm welfare risks using the ClassyFarm protocol and to identify if welfare risk changed according to farm size.

## 2. Materials and Methods

The study was carried out on 12 semi-extensively dairy sheep farms located in south-eastern Tuscany. The considered area has a Mediterranean climate type, with average temperatures ranging from 3 to 9 °C in winter and from 21 to 24 °C in summer. The rainfall is concentrated in autumn and in the period between winter and spring. The average rainfall is about 500 mm per year, distributed over 66 days of rain/year. The farms were selected and classified according to the herd size in agreement with the classification of the National Register of Livestock for the region and province included in the study [14]. Farms were divided into: <500 (small farms; n = 5), from 500 to 1000 (medium farms; n = 4), and >1000 (large farms; n = 3) head. The welfare assessment of the farms was performed by two trained veterinarians and with the support of one expert livestock technician using the ClassyFarm checklist for dairy sheep. The checklist is divided into five areas: A—“Farm management and personnel”; B—“Structures and facilities”; C—“Animal-based measures”; D—”Emergency plan and alarm system”; and BIO—“Biosecurity”. Items in the ClassyFarm system’s checklists include two or three answer options: insufficient or acceptable or excellent; and insufficient or acceptable. Where only two answers are provided, in the tables the third is indicated as not applicable (na). The main items within each area and the evaluation criteria are defined in the checklist and available as Supplementary Materials (Supplementary Tables S1–S5).

The assessment (e.g., insufficient, acceptable, or excellent) for each item was carried out according to the flock size (small-, medium-, and large-sized farms). The results are reported in the tables as the percentage of the farms for each category (small-, medium-, and large-sized farms) that obtained that assessment.

In order to highlight any association between farm size and assessment of welfare risk, statistical analyses were carried out using the chi-square test by the JMP software [15]. The significance threshold was set at *p* < 0.05.

## 3. Results and Discussion

### 3.1. Farm Management and Personnel—Area A

The results of area A, with the stratification based on flock size, are shown in Table 1. According to these data, the number of operators was always insufficient (A.1), irrespective of the number of animals. The low number of workers employed is also in agreement with the reports of other authors in goat farms [16].

Moreover, the different-sized farms had different ratings (*p* < 0.001) regarding animal management (A.3). In particular, 67% of the farms with number of head > 1000 had an excellent rating regarding animal management (A.3), since compared to small and medium farms, they had a more accurate division into groups of animals based on the physiological phase (Table 1).

Better management of the groups based on the physiological needs of the animals was associated with a better management of nutrition (A.7). In fact, A.7 was found to be significantly associated (*p* < 0.001) with the flock size and all the farms with more than 1000 animals used a feed expert and had controlled grazing management. The results also showed that the size of flock was associated (*p* < 0.001) with a worse assessment of the daily frequency of animal inspections (A.4) and the cleaning of the drinkers (A.10). The importance of cleanliness of water is linked to the freedom from prolonged thirst since dirty water often reduces palatability [17].

In addition to the greater difficulties in managing a larger number of animals, the worsening of A.4 and A.10 items was also probably linked to the fact that the large farms have more automated operations. As regards the management and hygiene of milking operations (A.12), the assessments were significantly (*p* < 0.001) associated with the farm size and most of the farms showed insufficient values. This verification element requires the identification of adequate udder hygiene and the use of pre-/post-dipping procedures. The absence of these practices in sheep farms is previously reported in European flocks [18], unlike USA producers, who have implemented such practices in their milking routine [19]. Indeed, the proper implementation of milking operations and the use of post-dipping is one of the management strategies to be used to maintain udder health and to control subclinical mastitis [20].

The hygiene, cleaning, and management of housing environment and litter (A.11) assessments differed (*p* < 0.001) according to the farm size and appeared to be better on medium-sized farms, which showed optimal evaluations in 100% of cases. The frequency of changing the animals bedding also seems to influence milk production, since environmental conditions can affect udder health [16,20].

According to Dwyer [21], sheep welfare is also affected by the experience and attitude of the stockperson caring for them. Farmer attitude can also affect management decisions, which may have an indirect impact on the welfare of their sheep. For example, farmer attitudes towards sick or injured animals are related to treatment decisions. In this study the management of sick or injured animals (A.5), the type of handling (A.6) were all acceptable without differences between the categories of farms, therefore animal received appropriate treatment and the handling to send the animals for milking or to change the grazing area was appropriate.

The management of lambs until weaning (A.8) was excellent in all the farms, since they were always kept with their mother.

### 3.2. Structures and Facilities—Area B

Table 2 shows the results of the structures and facilities on the farms in the study.

Although the buildings and housing areas were always evaluated as “acceptable”, as they did not present dangers for animals (B.14), the farm size was significantly (*p* < 0.001) associated with the assessments of the presence of sufficient shelters in grazing and outdoor areas (B.15); specifically, 100% of small and 75% of medium farms were found to be acceptable for B.15.

While 100% of farms with fewer than 500 animals were evaluated as “acceptable”, this percentage decreased to 75% on farms with between 500 and 1000 animals and to 33% on farms with more than 1000 animals. Furthermore, other authors have reported that in the semi-extensive farming system, shelters are provided by the natural shadows of trees and shrubs in the pastures, and the shelters are often not adequate for a high number of animals [22]. The presence of shelter is especially relevant in the areas characterized by hot summers. Thus, if flocks are not suitably sheltered against high air temperatures and solar radiation, a major environmental threat to animal welfare is heat stress [23]. Sheep use behavioral mechanisms, such as seeking shelter or shade, as part of their ability to adapt to thermal extremes. Sheep are able to maintain body temperatures even at high ambient temperatures with the provision of shade [24,25]. A study of hill sheep reared in Northern Europe reported that the sheep in full fleece seek shelter only when they are outside their thermoneutral zone, which can occur infrequently in a temperate climate [26]. However, shorn sheep, and those with thin fleeces, do make more use of shelter, particularly on windy days. Furthermore, the provision of shelter has significant benefits for animal welfare and productivity [27].

Good milking practices should take into account dairy hygiene, as well as good functioning of milking machines [23]. Significant differences (*p* < 0.001) in the maintenance of milking systems were found according to the farm size (B.21). In fact, the large farms carried out periodic checks and specialist maintenance only in the case of equipment breakdowns, rather than scheduled and thorough maintenance. In contrast, the small and medium farms performed scheduled and thorough maintenance (40% of farms with less than 500 head, and 25% of farms with between 500 and 1000 animals).

The surface for the decubitus of adult animals indoors (B.17) was significantly (*p* < 0.001) associated with the flock size and was insufficient in most of the farms; in particular, in 80% of the small farms and in 67% of the large farms, there was a surface/head ratio less than 1.5 m^2^. Reduced space is associated with decreased activity and lying time [28,29], and a decreased immune response to challenge [30]. Furthermore, Napolitano et al. [31] reported that the most critical aspects in sheep dairy farming were the low space allowance. However, these aspects may be compensated for by the frequent access to the pasture.

A sufficient number of places in the feeder helps to reduce competition for feeding. The availability of the feed front (B.18), as well as the number and functioning of the drinkers (B.19), were significantly (*p* < 0.001) related to the flock size and found to be better on the small farms, in which the number of feed and water places was adequate.

All farms paid attention to the microclimate (B.22), lighting (B.23), and the areas used as infirmaries (B.20), whereas in a previous study, only 27% of farms had infirmaries [22].

### 3.3. Animal-Based Measures—Area C

Table 3 shows the results of the animal-based measures.

The results for item C.24 (relationship test between humans and animals) were acceptable regardless of the dimension of the farms. The withdrawal from the social group is an indicator of animals in chronic pain [17]. In fact, sheep are social animals and are highly motivated to remain within the social group. In this regard, the results for item C.25 (presence of isolated animals with lack of social contact) were acceptable regardless of the dimension of the farms.

The farm size was associated (*p* < 0.001) with the cleanliness of the animals (C.27): large farms obtained an excellent rating, probably due to better group management (A.3). Similarly, an association was found between flock size and the geometric mean of somatic cell count lower than 750,000/mL (evaluated as excellent) (*p* = 0.028; C.30). An increasing trend in excellent assessments from small to large farms was found. Regarding skin lesions (C.28), they were significantly (*p* < 0.01) associated with the flock size. The total of 100% of excellent assessments found in medium and large farms suggests that, for both pasture and indoors, the environment did not have injurious features. Marcone et al. [32] and Napolitano et al. [31] also reported a low number of skin lesions in semi-extensively reared sheep.

The assessment of annual mortality of adult sheep (C.31) and lambs (C.32) was found to be related to farm dimension (*p* < 0.001) and was excellent in medium and large flocks. According to Richmont et al. [17], lamb mortality may function as an “iceberg” indicator for more than one discomfort condition. In general, adequate maternal nutrition has been extensively demonstrated to be essential for lamb survival. Undernourished ewes produce lambs having a low birth weight, with impaired neo-natal behavior and poor ability to thermoregulate; undernourished ewes also have reduced expression of maternal behavior and a lower availability and quality of colostrum. Overweight ewes are also at risk of metabolic disorders and increased lamb mortality [33].

In agreement with Gastaldo et al. [22], our data highlight an excellent nutrition status of sheep evaluated by the body condition score (BCS) (C.26) (less than 5% of animals with BCS below or over the ideal). Maintaining ewes with an adequate body condition score correlates positively with most production traits [34].

Lameness (C.29) was also almost absent and effectively kept under control on all types of farms, but not the optimal stocking density (Table 2, B.17). In fact, high stocking density represents a predisposing factor for lameness, along with wet and humid substrates with sharp stones in the pasture, and dirty floors [35]. Lame ewes tend to spend less time grazing and feeding, and they compete less for feed, and this can impair their productive performance, including in terms of milk yield [35]. The absence of lameness can be related to the hygiene of environments, humidity and temperature control, and good food management found in this study. Low prevalence of lameness was also reported by Marcone et al. [32] in sheep flocks, which was also linked to dry pasture and bedding material condition.

In the evaluated farms, invasive animal practices (C.33) were absent. This is in contrast to what has been reported in meat or dual-purpose (meat and wool) sheep in Australia and South America [5], where tail docking is a common management practice in order to reduce the risk of flystrike. However, short docked tails are associated with welfare risks such as rectal prolapse and bacterial arthritis.

### 3.4. Emergency Plan and Alarm System—Area D

The results relating to the emergency plans and alert systems are reported in Table 4.

The only significant differences (*p* < 0.001) were related to the daily inspection of mechanical or automatic systems, including the milking system (D.37), and to the origin of the drinking water (*p* < 0.001; D.34). Unlike small and medium farms, where controls for D.37 were acceptable and carried out daily, with the possibility of making up for the lack of electricity with the use of a generator, 33% of large farms were assessed as being insufficient, did not have a generator, and thus did not have a way of safeguarding the health and well-being of animals in emergency conditions. This can be aggravated by the lower number of staff in relation to the number of animals (A.1).

Lastly, since there is a relationship between ready access to water and the absence of prolonged thirst, the presence of an alternative source of drinking water (D.34) was significantly (*p* < 0.001) associated with the farm size and evaluated as excellent in 60–75% of the small and medium farms, respectively, while in the large farms there was less focus on the lack of water.

### 3.5. Biosecurity—Area BIO

Table 5 shows the results regarding biosecurity according to flock size.

Biosecurity is defined as “the set of managerial and physical measures designed to reduce the introduction, establishment and spread of animal diseases, infections or infestations to, from and within an animal population” [35]. A biosecurity program improves animal welfare by keeping more animals healthy and resistant to environmental factors; it also serves to provide early detection of diseases and to control or eradicate diseases. Furthermore, it reduces the costs of treating diseases, with benefits in terms of productivity, profitability, food safety [36,37], and environmental impacts in relation to chemical residues [37]. In addition, biosecurity measures directly reduce the risk of zoonotic pathogen transmission to humans [38].

Some recommended on-farm biosecurity measures include restrictions on sharing and disinfection procedures for equipment, vehicles and facilities, tick and pest control, vaccination, movement controls and quarantine of newly introduced animals, culling of diseased animals, and disposing of carcasses [38].

In this study, farms used at least four biosecurity measures; in fact, BIO.4, BIO.7, BIO.9, and BIO.14 (Table 5) showed at least sufficient evaluations in all the farms. However, the data highlight that little attention was paid to the other biosecurity measures.

Other studies have also found that the adoption of biosecurity practices on dairy farms is limited worldwide [39,40,41], and that most rural livestock producers have a poor understanding of biosecurity [40,41]. In contrast, biosecurity plans mainly cover poultry and pig farms [41].

In general, the flock size was significantly (*p* < 0.001; BIO.1) associated with the assessments of the control plan for rats and flies. Only 33% of the farms with more than 1000 head were found to be acceptable, compared to small and medium farms, which were acceptable in 60 and 75% of the cases, respectively.

Health monitoring (BIO.12), and the control and prevention of udder infections (BIO.13), was significantly related to the farm dimension (*p* = 0.049; *p* < 0.001 respectively). Furthermore, BIO.12 and BIO.13 were not adequately managed by any of the three categories of farms. On the contrary, the control and prevention of endo/ectoparasitosis (BIO.14) showed different assessments (*p* < 0.001) according to the size of the flock and were acceptable in all of the farms. Our findings were in agreement with the reports on goats [16] and sheep farms from South Africa [42], where external parasite control or deworming is the main biosecurity practice implemented. In fact, grazing animals are exposed to endo and ectoparasitic diseases, which leads to a reduction in feeding efficiency, growth rate, and production performances, together with hair and skin lesions (Otranto and Lia, cited by Caroprese et al., [23]).

The BIO.11 assessments were associated (*p* < 0.001) with the farm size, and none of the large farms was aware of their epidemiological situation regarding the main sheep infectious diseases (paratuberculosis; VISNA MAEDI virus; contagious ovine digital dermatitis) in terms of their control and prevention (BIO.11). Therefore, most of the farms did not implement specific prevention plans, while only a small number of the small farms had an optimal management.

A similar trend was also found in relation to the control and prevention of udder infections (BIO.13), which were never carried out on farms with more than 500 animals.

Parameters BIO.5 and BIO.6 are related to the disinfection of vehicles upon entering the farm and the possibility of contact between vehicles and animals, and were also significantly (*p* < 0.001) associated with the farm size. In particular, farms with more than a thousand head were mostly found to be totally insufficient for BIO.6. These results are also probably due to the greater difficulty in managing the external spaces adjacent to the stables.

## 4. Conclusions

This report provides insight into risk categorization of the welfare in semi-extensive dairy sheep farms. All the farms included in the study show an adequate rating. However, some major critical issues emerged concerning the low ratio between the number of stockpersons and the number of animals within the farm, the small decubitus areas, poor udder cleaning procedures during milking operations, and, in terms of biosecurity, the lack of prevention, control, and eradication plans for the most important infectious ovine diseases. The results show that different items in the five areas evaluated, including the assessments of management of the flock, the number of daily inspections, and the hygiene of the water supplies, are associated with the farm size. In large farms, the assessment of the management of the flock was better, but the number of daily inspections and the hygiene of the water supplies were worse. This suggests that a higher number of animals is probably associated with a lower ability to control them and their environment.

## Figures and Tables

**Table 1:** Assessment of main items area A “Management and personnel”.

Area A	Flock Size										
Item	<500 Heads			between 500 and 1000 Heads			>1000 Heads				
	I	A	E	I	A	E	I	A	E	χ^2^	*p*
	% of the Farms			% of the Farms			% of the Farms				
A.1 Number of stockpersons	100	0	0	100	0	0	100	0	0	0.00	1.000
A.2 Farmer training	0	40	60	0	50	50	0	67	33	14.94	0.001
A.3 Management of groups of animals	0	100	0	0	75	25	0	33	67	107.83	<0.001
A.4 Frequency of animal inspections	0	40	60	0	50	50	0	100	0	88.99	<0.001
A.5 Management of sick animals	0	100	0	0	100	0	0	100	0	0.00	1.00
A.6 Type of handling	0	100	na	0	100	na	0	100	na	0.00	1.00
A.7 Management of feed	0	20	80	0	50	50	0	0	100	70.81	<0.001
A.8 Management of lambs	0	0	100	0	0	100	0	0	100	0.00	1.00
A.9 Water availability	40	0	60	0	100	0	0	100	0	92.31	1.00
A.10 Cleanliness of drinkers	0	40	60	0	0	100	0	67	33	99.05	<0.001
A.11 Hygiene, of housing	0	40	60	0	0	100	0	33	67	49.56	<0.001
A.12 Hygiene of milking operations	80	20	0	75	25	0	100	0	0	27.45	<0.001

na = not applicable; I = Insufficient; A = Acceptable; E = Excellent.

**Table 2:** Assessment of main items of area B “Structures and facilities”.

Area B	Flock Size										
Item	<500 Heads			between 500 and 1000 Heads			>1000 Heads				
	I	A	E	I	A	E	I	A	E	χ^2^	*p*
	% of the Farms			% of the Farms			% of the Farms				
B.14 Farm structures	0	100	na	0	100	na	0	100	na	0.00	1.00
B.15 Presence of shelter	0	100	0	25	75	0	67	33	0	107.83	<0.001
B.16 Type of animal housing	0	0	100	0	0	100	0	0	100	0.00	1.00
B.17 Surface for the decubitus	80	20	0	50	0	50	67	0	33	93.62	<0.001
B.18 Number of places available in the feeder	0	60	40	25	50	25	33	67	0	70.82	<0.001
B.19 Size and operation of drinkers	20	60	20	75	25	0	67	33	0	89.80	<0.001
B.20 Infirmary	0	80	20	0	100	0	0	67	33	38.00	<0.001
B.21 Milking system maintenance	0	60	40	0	75	25	0	100	0	48.12	<0.001
B.22 Temperature and humidity	0	20	80	0	25	75	0	0	100	27.45	<0.001
B.23 Artificial lighting	0	100	na	0	100	na	0	100	na	0.00	1.00

na = not applicable; I = Insufficient; A = Acceptable; E = Excellent.

**Table 3:** Assessment of main items of area C “Animal based measures”.

Area C	Flock Size										
Item	<500 Heads			between 500 and 1000 Heads			>1000 Heads				
	I	A	E	I	A	E	I	A	E	χ^2^	*p*
	% of the Farms			% of the Farms			% of the Farms				
C.24 Relation test	0	100	na	0	100	na	0	100	na	0.00	1.00
C.25 withdrawal from the social group	0	100	0	0	100	0	0	100	0	0.00	1.00
C.26 Nutrition status	0	0	100	0	0	100	0	0	100	0.00	1.00
C.27 Cleanness of animals	20	20	60	0	25	75	0	0	100	73.76	<0.001
C.28 Skin lesions	0	20	80	0	0	100	0	0	100	42.86	<0.001
C.29 Prevalence of lameness	0	0	100	0	0	100	0	0	100	0.00	1.00
C.30 Udder health	40	40	20	50	25	25	33	33	34	10.83	0.028
C.31 Annual mortality of adult sheep	20	0	80	0	0	100	0	0	100	42.86	<0.001
C.32 Annual lamb mortality	40	0	60	0	0	100	0	0	100	92.31	<0.001
C.33 Mutilations	0	0	100	0	0	100	0	0	100	0.00	1.00

na = not applicable; I = Insufficient; A = Acceptable; E = Excellent; significance *p* < 0.05.

**Table 4:** Assessment of main items of area D. emergency plans and alert systems according to flock size.

Area D	Flock size										
Item	<500 Heads			between 500 and 1000 Heads			>1000 Heads				
	I	A	E	I	A	E	I	A	E	χ^2^	*p*
	% of the Farms			% of the Farms			% of the Farms				
D.34 Origin of the drinking water	20	20	60	0	25	75	34	33	33	51.93	<0.001
D.35 Lighting	0	100	na	0	100	na	0	100	na	0.00	1.00
D.36 Fire alarm	100	0	na	100	0	na	100	0	na	0.00	1.00
D.37 Inspection of equipment	0	100	0	0	100	0	33	67	0	74.16	<0.001
D.38 Treatment register	0	100	na	0	100	na	0	100	na	0.00	1.00
D.39 Register for loading and unloading animals	0	100	na	0	100	na	0	100	na	0.00	1.00
D.40 Illicit substances	0	100	na	0	100	na	0	100	na	0.00	1.00

na = not applicable; I = Insufficient; A = Acceptable; E = Excellent; significance *p* < 0.05.

**Table 5:** Assessment of biosecurity (BIO) according to flock size.

Area BIO	Flock size										
Item	<500 Heads			between 500 and 1000 Heads			>1000 Heads				
	I	A	E	I	A	E	I	A	E	χ^2^	*p*
	% of the Farms			% of the Farms			% of the Farms				
BIO.1- Control plan for rats and flies	20	60	20	25	75	0	67	33	0	91.87	<0.001
BIO.2 Contact with other animal species	60	40	0	75	25	0	67	33	0	51.12	0.077
BIO.3 Precautions for the entry of unknown visitors	20	80	0	75	25	0	0	67	33	190.09	<0.001
BIO.4 Management of regular visitors	0	100	0	0	100	0	0	100	0	0.00	1.00
BIO.5 Disinfection of vehicles	0	100	0	25	75	0	33	67	0	38.00	<0.001
BIO.6 Contact between visitors’ vehicles and animals	80	20	na	75	25	na	100	0	na	27.45	<0.001
BIO.7 Collection of carcasses	0	100	na	0	100	na	0	100	na	0.00	1.00
BIO.8 Loading of live animals	80	20	na	100	0	na	100	0	na	42.86	<0.001
BIO.9 Animal movement	0	80	20	0	100	0	0	67	33	38.00	<0.001
BIO.10 Quarantine/settlement management	40	60	0	50	50	0	33	67	0	6.04	0.049
BIO.11 Control of the main infectious diseases	80	0	20	75	25	0	100	0	0	96.12	<0.001
BIO.12- Health monitoring	60	40	na	50	50	na	67	33	na	6.04	0.049
BIO.13 Control of udder infections	80	0	20	100	0	0	100	0	0	42.86	<0.001
BIO.14 Control of endo/ectoparasitosis	0	20	80	0	50	50	0	100	0	133.03	<0.001
BIO.15 Control of water sources	40	60	na	50	50	na	0	100	na	66.67	<0.001

na = not applicable; I = Insufficient; A = Acceptable; E = Excellent; significance *p* < 0.05.

## Data Availability

The data presented in this study are available on request from the corresponding author.

## Supplementary Materials

The following supporting information can be downloaded at: https://www.mdpi.com/article/10.3390/ani14101401/s1, Table S1: Main items and specific thresholds for Area A: Farm management and personnel; Table S2: Main items and specific thresholds for Area B: Structures and facilities; Table S3: Main items and specific thresholds for Area C: Animal-based measures; Table S4: Main items and specific thresholds for Area D: Emergency plan and alert system; Table S5: Main items and specific thresholds for Area BIO: Biosecurity.
