# Supplementary material for: Risk Categorization in On-Farm Welfare in Different-Sized Dairy Sheep Flocks
Source: Animals (Basel). 2024 May 7;14(10):1401. doi: 10.3390/ani14101401 (PMC11117197; doi:10.3390/ani14101401)
Supplement: Supplementary file 1 [file animals-14-01401-s001.zip › animals-2912355-supplementary.pdf]

Table S1. Main items and specific thresholds for Area A: Farm management and personnel.

Item	Item description	Insufficient	Acceptable	Excellent
A.1 Number of stockpersons	N of operators involved in the management of the animals	✓ one for more than 300 animals[1]	✓ one for 200-300 animals[1]	✓ one for less than 200 animals[1]
		✓ one for more than 150 animals[2]	✓ one for 100-150 animals[2]	✓ one operator for less than 100 animals[2]
		✓ one for more than 100 animals[3]	✓ one for 60- 100 animals[3]	✓ one for less than 60 animals[3] one for less than 100 total animals[4]
A.2 Farmer training	years of experience and training	<7 years and no training	At least 7 years and no training	At least 7 years and educational qualification or relevant training within the last 3 years
A.3 Management of the groups of animals	For the correct management of the specific needs at least the following categories need to be present: lambs (younger than 3 months), replacement lambs, dry sheep and lactating sheep, rams	Number of groups not corresponding to those expected	Same number of groups as expected	Multiple groups (i.e. primiparous, pluriparous, early-late lactation) and controlled management of rams
A.4 Frequency of animal inspection	Number of daily visits outside of the routine activities (milking and feeding)	HOUSING: less than one inspection/day<br>PASTURE: inspection at insufficient intervals	HOUSING: one or more inspections/day without written reports<br>PASTURE: Inspection at sufficient intervals	HOUSING/ PASTURE: two or more inspections/day and written reports or computerized logging
A.5 Management of sick animals	Appropriate treatment of sick or injured animals and presence of trained staff	Untrained personnel and/or animals that need treatment and have not received it and/or the absence of farm veterinarian	Trained staff with evidence of animals in the infirmary or treatments in progress and the presence of farm veterinarian	In addition to the adequacy criteria, the presence of written procedures for the treatment of animals
A.6 Type of handling	Correct handling to send the animals for milking or to change the grazing area	Use of potentially harmful tools (electric prods and/or sharp instruments)	Use of non-injurious tools (voice, hands and/or flexible plastic rods)	na
A.7 Management of feed and daily ration	Presence of specific rations for dry ewes, replacement, lactation and rams	HOUSED: i.e. empirical ration or composed of unhealthy foods<br>PASTURE: i.e. no grazing techniques and no additional integration	HOUSED: i.e. specific ration for each group<br>PASTURE: i.e. diet based on rotational grazing and food supplementation in certain periods	HOUSED: i.e. ration calculated by a nutritionist<br>PASTURE: i.e. controlled management of grazing times and herbaceous essences
A.8 Management of lambs until weaning		✓ Lamb removed from its mother<br>✓ fed with milk substitute at a unsuitable temperature<br>✓ in case of automatic lamb feeder in operation for more than 24 hours=1 teat for more than 25 animals	✓ Lamb removed from its mother<br>✓ fed with milk substitute at a suitable temperature twice a day<br>✓ in case of automatic lamb feeder in operation for 24 hours =1 teat 25 animals	Lamb kept with its mother until weaning

A.9 Water availability		HOUSED Absence of drinking water or rationed water<br><br>PASTURE: excessive distance between water sources, unhealthy water	HOUSED functioning drinkers in all groups, in sufficient numbers and clean water<br>PASTURE: functioning drinkers in all groups, near grazing areas, or mobile drinkers	Ad libitum water for all animals and water given (in addition to the milk at least once a day) also to the lambs before weaning
A.10 Cleanliness of drinkers		Dirt on the surface and on the walls of the drinkers	Food residues only on the surface	Clean drinkers and clear water
A.11 Hygiene of housing	Cleaning and management of housing environments and litter	Dirty, unmanaged and/or harmful to the animals housing and bedding areas	Fairly clean and sufficiently managed housing and litter areas	Clean and dry housing and bedding environments, optimally managed
A.12 Hygiene of milking operations	Udder hygiene	Lack of udder hygiene	Adequate cleaning of the udder,<br>use of non-routinely pre/post dipping	Adequate cleaning of the udder,<br>routine use of pre/post dipping with sprays or clean glasses

[1]the milking is performed by others; [2]the same person also performs the milking; [3]the same person also performs hand milking; [4]if the same person also performs the milking; na=not applicable.

Table S2. Main items and specific thresholds for Area B: Structures and facilities.

Item	Item description			
		Insufficient	Acceptable	Excellent
B.14 Farm buildings	Harmfulness to animals of housing areas, environments or equipment	harmful	not harmful	na
B.15 Presence of shelter on the pasture		Absence, insufficiency or inadequate shelters	Sufficient and adequate shelters for all animals	Easily accessible artificial shelters, sufficient and suitable for protection from environment
B.16 Type of animal housing		Conventional barns, kept tight for a group of animals	Loose housing for all groups of animals	Loose housing for all animals and possibility for adult sheep to access an exercise area with a surface area of at least 1.7 m^2/head
B.17 Surface for decubitus	Freedom of movement Surface available for adult sheep	Limited: surface area of less than 1.5 m^2/head	Ensured: a surface of between 1.5 - 1.7 m^2/head	Free-range animals with an available area exceeding 1.7 m^2/head as well as the possibility of accessing a suitable exercise or grazing area
B.18 Number of places available in the feeder	Access to food for adult sheep	places in the feeding trough or unitary front with spaces smaller than the number of animals	places in the feeding trough or unitary front with spaces equal to the number of animals	places in the feeding trough or unitary front with spaces greater than the number of animals or suitable pasture
B.19 Size and operation of drinkers		less than 1 drinker every 25 heads	1 drinker every 25 heads	More than 1 drinker every 25 heads and with diversified locations in each area of the farm
B. 20 Infirmary	Presence of a room in which injured or sick animals can be isolated	Absence	Specific room equipped, where appropriate, with dry and comfortable litter	Loose animals in a specific and identified room, with dry and comfortable permanent litter with suitable dimensions (able to accommodate 3% of the average number of animals present in the farm)
B.21 Milking system maintenance	Periodic checks of milking system	Absence of scheduled maintenance	Not well-executed periodic checks, specialized maintenance only in case of breakdowns and no documentation certifying periodic maintenance	Scheduled maintenance, periodic checks and availability of a written records
B.22 Temperature and humidity	Microclimatic conditions	Harmful: i.e. closed or dusty environments or semi-open stables without adequate ventilation	Suitable: i.e. natural ventilation (open barn) or suitable ventilation/ventilation systems or the possibility of modulating the openings according to the climate	Optimal: i.e. conditioning systems with insulation systems
B.23 Artificial lighting	Natural or artificial	Insufficient	Adequate natural or artificial lighting for at least 8 hours/day	na

na=not applicable.

Table S3. Main items and specific thresholds for Area C: Animal-based measures.

Item	Item description			
		**Insufficient**	**Acceptable**	**Excellent**
C.24 Relationship test between humans and animals (adult sheep)		The animals leave when the farmer arrives	The animals remain still or approach the farmer upon arrival	na
C.25 Withdrawal from the social group	% of isolated animals	More than 3%	Between 1-3%	Less than 1%
C.26 Nutrition status (adult sheep)	% of the animal with BCS beyond the accepted limits: less than 2 (very lean animals) and greater than 4 (very fat animals)	More than 10% over the limits	Between 5 and 10% over the limits	Less than 5% over the limits
C.27 Cleanness of animals	State of cleanliness of the fleece (neck, abdomen, udder, limbs and tail)<br>% of dirty animals	More than 20%	Between 7% and 20%	Less than 7%
C.28 Skin lesions	% of animals with skin lesions	More than 10% of animals	Between 5% and 10%	Less than 5%
C.29 Prevalence of lameness (adult sheep)	% of animal lame	More than 10%	Between 6% and 10%	Less than 6%
C.30 Udder health	Geometric mean of somatic cell counts	greater than 1,500,000 cells/ml and/or no data in the last 3 months	between 750,000 and 1,500,000 cells/ml	less than 750,000 cells/ml
C.31 Annual mortality of adult sheep	Number of sheep that died spontaneously, emergency slaughtered, euthanized in the last 12 months compared to the number of adult sheep present on the day of the visit	More than 5%	Between 2% and 5%	Less than 2%
C.32 Annual lamb mortality<br><br>s	Number of lambs that died from the 2nd to the 90th day of life, excluding stillbirths, compared to the total number of lambs born alive and viable in the last 12 month	More than 10%	Between 4 and 10%	less than 4%
C.33 Mutilations	incisions or with mutilations/castrations	without respecting the times and methods established by law	performed in compliance with the times and methods established by law	no incisions or mutilations/castrations

na=not applicable.

Table S4. Main items and specific thresholds for Area D: Emergency plan and alert system

Item	Item description			
		**Insufficient**	**Acceptable**	**Excellent**
D.34 Origin of the drinking water	Number of sources of water (well, natural source or aqueduct), presence of cistern	Only from one source	From an aqueduct or well with a large cistern with sufficient water for a few hours	Multiple sources
D.35 Lighting	Artificial lighting for inspection	Absent	Correct and functioning artificial lighting	na
D.36 Fire alarm (or prevention systems)	Fire alarm or prevention systems	Absent	Present	na
D.37 Inspection of automatic and mechanical equipment	Every automatic or mechanical plant essential for animal health (i.e. milking machine) must be inspected once a day and any defects found eliminated immediately. Adequate measures must be taken to safeguard the health and welfare of the animals (i.e. power generator)"	Lack inspections or of timely interventions	At least one inspection per day	Procedures or good practice manuals with operating instructions, and records
D.38 Treatment register	Maintenance of the register	Absent or non-compliant	Present and compliant	na
D.39 Register of loading and unloading of animals	Maintenance of the register	Absent or non-compliant, o abnormal mortality	Present and compliant	na
D.40 Administration of illicit substances		Non-compliant	Compliant	na

na=not applicable.

Table S5. Main items and specific thresholds for Area BIO: Biosecurity.

Item	Item description			
		**Insufficient**	**Acceptable**	**Excellent**
BIO.1 Control plan for rats and flies	Presence of procedures and control plans	No procedures	Non-formalized procedures	written procedures and/or biosafety manual
BIO.2 Contact with other animal species	Frequency of contact with other animal species	Frequent and obvious	Possible but it is not evident	Contact cannot occur. The farm is well protected (fences, etc.); there are no other species of animals within the farm perimeter
BIO.3 General precautions for unknown visitors	Presence of precautions procedures for unknown visitors	No procedures	Non-formalized procedures	Procedures and/or written in a biosafety manual
BIO.4 Management of regular visitors	Precautions regarding the clothing	No precautions	All "visitors" must wear disposable shoes before entering the stables	All "visitors" must pass through a changing room area and must wear disposable socks and gowns
BIO.5 Disinfection of vehicles upon entering the farm	Presence of disinfection aids/devices	Absence	Non-specific disinfection aids used only in case of need	Specific, fixed and routinely used disinfection aids
BIO.6 Possibility of contact between foreign vehicles and reared animals		Yes	No	na
BIO.7 Collection of carcasses-	The vehicle for the collection of dead animals cross areas that allow contact (< 20 m) with areas where there are reared animals	Yes	No, the vehicle is blocked at the confines of the farm, where the carcass/s was/were previously collected	na
BIO.8 Loading of live animals	Loading of live animals close or far from the stables where the animals are reared	Close to the stables (<20 m)	Away from the stables (>20 m)	na
BIO.9 Animal movement	Frequency of incoming and outcoming animals	Routine outcoming animals (exhibitions/markets/pasture with other herds)	Purchase/incoming only in case of emergency or need (in the last 2 years) and no movement of animals	No incoming and no handling of animals for over 2 years
BIO.10 Quarantine / settlement management	Quarantine, times structures, and biological tests on the animals purchased	No form of quarantine	Partial quarantine, carried out in an empirical way (dedicated area adjacent to the rest of the animals / short times)	No incoming of animals for over 2 years, or correct form of quarantine, in terms of times and structures and completed with biological tests

BIO.11 Control and prevention of the main infectious diseases	Knowledge, presence of control and prevention plans (vaccination, elimination of infected animals, eradication plans, etc.) of paratuberculosis; MAEDI VISNA virus; contagious ovine digital dermatitis	No knowledge or no information on the health status of the herd	Partial knowledge and/or presence of undefined plans	✓ Knowledge of the 3 pathologies and of the relative situation / prevalence on the farm<br>✓ application of correct operational plans on at least 2 of them,<br>✓ prevention and control
BIO.12 Health monitoring activities-	Farm's habit of delivering biological and/or pathological material (i.e. faeces, nasal mucus, saliva, blood samples, fetuses, etc.) to the laboratory of reference analysis for health investigations/monitoring	Absence	Evidences of analytical result of the last 12 months	na
BIO.13 Control and prevention of udder infections-	Microbiological analytical results (in the last 12 months) on bulk milk or individual animal, such as to indicate the risk of mastitis	Absence	Only bulk analyses	Analyses of problematic animals and eradication or control plans
BIO.14 Control and prevention of endo / ectoparasitosis	Knowledge of the health status and prevention /control plans	Absence	Partial knowledge and/or presence of undefined plans	Knowledge of the main parasitosis; antiparasitic treatments planned and/or performed following laboratory tests
BIO.15 Control and analysis of water sources		No well water analysis	Well water analyses at least once a year / municipal aqueduct water	na

na=not applicable.
